# Research progress of electronic nose technology in exhaled breath disease analysis

**DOI:** 10.1038/s41378-023-00594-0

**Published:** 2023-10-11

**Authors:** Ying Li, Xiangyang Wei, Yumeng Zhou, Jing Wang, Rui You

**Affiliations:** 1 School of Instrument Science and Opto-Electronics Engineering, Beijing Information Science and Technology University, Beijing, 100192 China; 2 Laboratory of Intelligent Microsystems, Beijing Information Science and Technology University, Beijing, 100192 China; 3 School of Electronics and Information Engineering, Changchun University of Science and Technology, Changchun, 130022 China

**Keywords:** Electrical and electronic engineering, Sensors

## Abstract

Exhaled breath analysis has attracted considerable attention as a noninvasive and portable health diagnosis method due to numerous advantages, such as convenience, safety, simplicity, and avoidance of discomfort. Based on many studies, exhaled breath analysis is a promising medical detection technology capable of diagnosing different diseases by analyzing the concentration, type and other characteristics of specific gases. In the existing gas analysis technology, the electronic nose (eNose) analysis method has great advantages of high sensitivity, rapid response, real-time monitoring, ease of use and portability. Herein, this review is intended to provide an overview of the application of human exhaled breath components in disease diagnosis, existing breath testing technologies and the development and research status of electronic nose technology. In the electronic nose technology section, the three aspects of sensors, algorithms and existing systems are summarized in detail. Moreover, the related challenges and limitations involved in the abovementioned technologies are also discussed. Finally, the conclusion and perspective of eNose technology are presented.

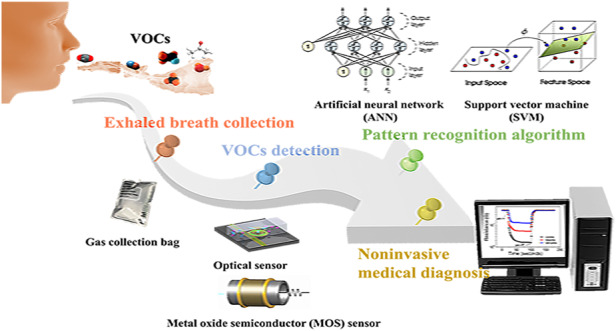

## Introduction

Human exhaled gas is composed of 150 mL of ‘dead space gas’ and approximately 350 mL of ‘alveolar gas’^1^. ‘Alveolar gas’ refers to the headspace gas of human blood, which can dynamically reflect the trend of blood metabolism^2^. Exhaled gases of healthy humans contain nitrogen, oxygen, carbon dioxide, water vapor, rare gases, and various compounds produced during metabolism^3–6^. These compounds contain trace amounts of volatile organic compounds (VOCs) and some nonvolatile components, usually between one trillionth (ppt) and one millionth (ppm)^7^. Various gases have different types, concentrations, volatilities, fat solubilities, diffusion rates in the blood circulation, passing rates through alveolar cell membranes, and other characteristics^8^. When one or more gas concentration exceed a certain range or some specific gases are produced, they often cause changes in the body’s disease or metabolic function^9–11^. Significant changes in breath markers can be detected in many diseases, among which Helicobacter pylori breath detection has become a clinical basis^12,13^, and exhaled NO detection can also be used as an auxiliary means of asthma clinical^14^.

As noninvasive medical diagnostic and therapeutic technologies continue to advance, exhaled breath analysis is the most likely alternative to noninvasive and portable health diagnosis. It has the advantages of being noninvasive, painless, safe and convenient, and simple operation. Moreover, it can also avoid the discomfort and embarrassment caused by blood and urine tests. In summary, breath analysis is a highly a promising medical detection technology^15–17^. Thousands of different gases contained in human exhaled breath are products of human metabolism and exposure to exogenous compounds. These exhaled breath biomarkers can characterize the effects of external factors on human health. By testing the relative levels of certain biomarkers, the health status of the human body can potentially be determined. The detection of human exhaled breath is usually based on mass spectrometry and gas chromatography. However, this related equipment is expensive, complicated to operate, and not portable enough, which limits its practical application in the field of breath diagnosis^18,19^. Unlike the traditional methods of testing human exhalation described above, the electronic nose (eNose) usually does not require expensive components or skilled operators. In addition, the operation time is relatively short, with results available in a few minutes.

eNose is an intelligent system that combines a cross-sensitive chemical sensor array with an effective set of pattern recognition algorithms to detect, identify or quantify various gases/odors. First, a series of gas-sensitive sensors with good resolution and selectivity to the target analytes are selected to form a sensor array. Then, the response curve of this sensor array is obtained through a data acquisition card to extract feature parameters after denoising of these response signals. Finally, the extracted feature parameters are fed into the pattern recognition system to identify the type and concentration information of the gas/odor. The utilization of eNose technology in noninvasively diagnosing human exhalation provides significant advantages, such as low technical costs and excellent discrimination capabilities.

With the continuous development of gas sensing technology and artificial intelligence, the human exhaled breath detection method based on eNose technology has the potential for large-scale early diagnostic screening and long-term monitoring and diagnosis. eNose technology has the advantages of miniaturization, easy integration, economic benefits, and simple operation. The development of eNose technology in the field of health care has greatly expanded^20^. The application of eNose in clinical medicine mainly includes early screening of various cancers^21^, lung diseases, such as pneumonia and upper respiratory tract infection^22^, diabetes^23^, identification of bacterial pathogens^24^, and microbial metabolites released from superficial wounds^25^.

After nearly three decades of development, eNose technology has made great progress. However, several challenges persist. One such challenge is the presence of the drift phenomenon, where the sensor response and pattern recognition algorithm (PRA) gradually deviate over time. This drift hinders the alignment between the sensor response and the algorithm’s performance, leading to decreased matching accuracy. Furthermore, the collected data from sensor arrays for the same detection target consist of multivariate time series signals with complex structures. In addition, a priori response functions and accurate mathematical models for gas-sensitive sensors are difficult to obtain due to the complexity of the response mechanism. Consequently, researchers still rely on empirical approaches when choosing signal processing and pattern recognition algorithms. These unresolved issues have impeded the widespread utilization and advancement of eNose technology. Therefore, exploring and researching solutions for real-time, fast, efficient, and accurate gas identification within the eNose domain remains an imperative research direction.

**Fig. 1:**
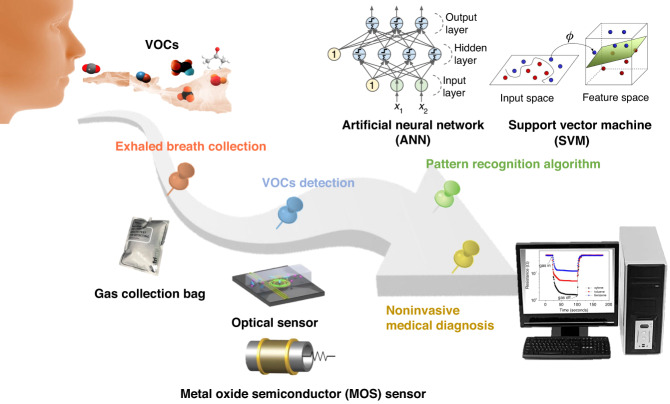
Schematic diagram of the noninvasive breath detection via the eNose system

Here, an overview and analysis of the research conducted on eNose technology for noninvasive breath diagnosis is presented. Its working schematic diagram is shown in Fig. 1. In this review, the significance of utilizing human exhaled breath as a diagnostic tool for various diseases is initially highlighted. The correlation between certain diseases and specific biomarkers present in human exhaled breath are elucidated. Then, several existing methods for detecting expiratory breath and their underlying principles are summarized and demonstrated. Through a comparative analysis of their practical advantages and limitations, the expiratory breath detection method based on eNose emerges as an ideal noninvasive diagnostic approach. In the subsequent section, the gas sensors and PRA used within the eNose system are two technological aspects that serve as crucial components, and each are thoroughly discussed. Then, the research progress of eNose technology for disease breath analysis is introduced, and the applications of eNose technology in this field are provided. Finally, the main challenges existing at present and the prospect of future development are presented.

## Application of human exhaled breath components in disease diagnosis

Exhalation is a process of gas exchange between the human body and the outside environment. It is one of the most important metabolic activities of organisms. Exhaled gas contains much information related to body health. In 1971, Linus et al. published a significant article in which more than 200 ppm levels of VOCs were detected in exhaled gas through gas chromatography^26^. This discovery paved the way for various methods of exhalation analysis. With the development of exhaled breath analysis and detection, the study of VOC biomarkers in human exhaled breath for metabolic diseases has attracted wide attention. Currently, more than 3000 different VOCs have been identified in breath samples^19,27–29^, with over 500 VOCs detected in single breath samples^27,30,31^.

In addition, inorganic and organic compounds have also been found in human exhaled breath. Inorganic compounds in human exhaled breath include nitric oxide (NO), carbon monoxide (CO), ammonia (NH_3_), and hydrogen sulfide (H_2_S). Organic compounds mainly include hydrocarbons (such as ethane, pentane, and isoprene), oxygen-containing compounds (such as acetone, alcohols, and aldehydes), nitrogen-containing compounds (such as dimethylamine and trimethylamine) and sulfur-containing compounds (such as methyl mercaptan, ethyl mercaptan, and dimethyl sulfide)^4,32–34^. The prevalent compounds detected in human exhaled breath are summarized in Table 1, as well as their corresponding disease types and exhaled breath concentrations observed in healthy people. These are expected to become potential biomarkers for disease diagnosis.

**Table 1:** Potential disease biomarkers in human exhaled breath

Biomarker	Disease	Exhaled concentration of healthy people
Nitric oxide	Asthma^7,53,159,160^, COPD^7^, cystic fibrosis^160^	~10 ppb^35^ ; ~30 ppb^36^; 10~50 ppb^161^
Carbon Monoxide	Asthma^162^, COPD^7^, airway inflammation^160^	0~6 ppm^161^; 1~2 ppm^162^
Carbon dioxide	Helicobacter pylori infection^163^	4~5%^160^
Methane	Intestinal malabsorption^164^, visceral fat accumulation^165^	2~10 ppm^161^
Ethane	COPD^2,7,160^, asthma^2,160^, ulcerative colitis^10^	0~10 ppb^161^
Pentane	COPD^2^, asthma^2,160^, cystic fibrosis^160^, breast cancer^46^, ulcerative colitis^10^	0~10 ppb^161^
Isoprene	LC^160^, cholesterol metabolism^166^	22~234 ppb^7^; Average 100 ppb^44^; ~105 ppb^161^; 12~580 ppb^166^
Acetone	Diabetes^18,52,53^	0.3~0.9 ppm^44,51,83^; 0.3~1 ppm^161^
Methanol	LC, cystic fibrosis^11^	~1 ppm^160^; 160~2000 ppb^166^
Ethyl alcohol	Cystic fibrosis, diabetes^11^	~1 ppm^160^; 13~1000 ppb^166^
Formaldehyde	LC^7,167^	Average 48 ppb^167^
Ammonia	Kidney disease^53,159^, liver disease^2,7,159^, asthma^159^, halitosis^7^	248~2935 ppb^7^; 425~1800 ppb^45^; 50~2000 ppb^47^; 0.5~2 ppm^161^
Hydrogen sulfide	Halitosis^52,53^	0~1.3 ppm^161^; 150 ppb^168^

**Fig. 2:**
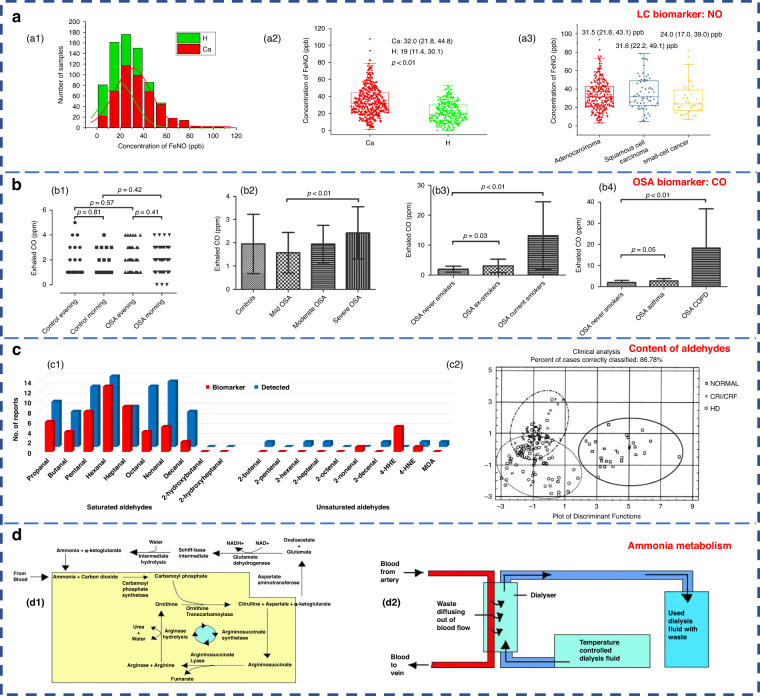
Application of human exhaled breath in disease diagnosis. **a** NO content in exhaled breath of H subjects (green) and LC patients (red)^35^. Copyright 2021 MDPI. **b** Different types of OSA in patients with exhaled CO content diagram^39^. **b1–b4** Four OSA patients with different degrees of physical health. Copyright 2017 the American Physiological Society. **c** Comparison of the content of aldehydes in the exhaled breath of H subjects and patients^55^. **c1** Statistical results of the detection of aldehydes in the exhaled breath of H subjects (blue) and LC patients (red). **c2** Exhaled breath samples of uremic hemodialysis (HD) patients (symbol ○), chronic renal insufficiency or chronic renal failure (CRI/CRF) patients (symbol ×) and H subjects (symbol □). Copyright 2022 MDPI. **d** Ammonia metabolism^55^. **d1** The urea cycle. **d2** Hemodialysis. Copyright 2011 Informa UK Limited

Inorganic compounds, such as NO, have been used as biomarkers of lung inflammation and have shown potential in the study of various lung diseases. Their clinical value for the diagnosis of patients with lung cancer (LC) is considerable^35^. As shown in Fig. 2a, breath samples were collected from healthy people (H) and LC patients. The H subjects exhibited a considerably higher count of individuals with exhaled breath NO levels below 20 ppb compared to the LC group. Furthermore, the H subjects demonstrated a maximum level of exhaled breath NO below 60 ppb, while the LC group showed a maximum level of exhaled breath NO surpassing 100 ppb. Exhaled NO detection has been approved by the U.S. Food and Drug Administration as a diagnostic criterion for asthma, thus positioning it as a valuable adjunctive tool for asthma assessment and treatment^36^. Exhaled CO may be associated with obstructive sleep apnea (OSA), a common sleep-disordered breathing disorder characterized by recurrent complete or partial collapse of the upper airway during sleep^37^. The resulting intermittent hypoxia can lead to airway inflammation and oxidative stress. Endogenous CO is mainly a byproduct of heme oxygenase-catalyzed heme degradation^38^. It is a marker of oxidative stress. Studies have shown elevated levels of exhaled circulating CO in patients with OSA^39^. The exhaled CO content in patients with different types of OSA is demonstrated in Fig. 2b.

Hydrocarbons are compounds derived from lipid peroxidation^40^ and can serve as biomarkers of oxidative stress^2^. Oxidative stress is the most frequent pathological state in major diseases such as asthma, chronic obstructive pulmonary disease (COPD) and LC. They can be characterized by chronic inflammation and oxidative stress, which can be diagnosed by endogenous volatiles^41,42^. Specifically, most of the VOCs in COPD are aldehydes or hydrocarbons^43^. The saturated aldehydes in the exhaled breath of patients with LC showed distinctive disparities compared to those of the H subjects (Fig. 2c1, red bars). Oxidative stress metabolites are considered to be the main components of abnormal exhaled breath in LC^44^. Additionally, hydrocarbons, such as methane, ethane and pentane, can serve as biomarkers for asthma, breast cancer, liver disease, and intestinal and colon-related diseases^34,45^. The exhaled breath of breast cancer patients contains volatile alkanes (such as pentane, hexane and long-chain alkanes) and alkane derivatives, which are derived from oxidative stress associated with breast cancer lesions^30^ or induced activation of polymorphic cytochrome mixed oxidase^46^. Isoprene is the main hydrocarbon found in human exhaled gas^34^ and is associated with cholesterol metabolism^45^.

Many studies have shown that acetone is one of the most abundant VOCs in human respiration^4,47–49^. The research results show that acetone in human exhaled breath can be used as the main characteristic marker of diabetes due to its high sensitivity and specificity^50^. Ketones in the human body are produced when the liver decomposes fat and are special intermediate products of fat metabolism. Among them, 3-β-hydroxybutyric acid and acetoacetic acid are not volatile; thus, the ketone present in exhaled air is mainly acetone. The concentration of acetone in the exhaled breath of diabetic patients can reach 2–6 times higher than that of the H subjects, as shown in Table 1^51–53^. Ethanol and methanol in the human body are derived from microbial fermentation of carbohydrates in the gastrointestinal tract^34,54^. Increased levels of reactive oxygen species in cancer cells promote lipid peroxidation, leading to the production of various aldehydes^55^. Therefore, the content of ethanol, ketones and aldehydes in the exhaled breath of cancer patients is significantly higher than that of the H subjects^56^. In addition, formaldehyde has also been proposed as a marker for LC^7^.

Ammonia is the main nitrogen-containing volatile compound. Abnormal levels of ammonia in breath are associated with liver or kidney dysfunction^57^, which could also be used to diagnose peptic ulcers of the stomach or duodenum caused by Helicobacter pylori^58^. Additionally, elevated concentrations of dimethylamine and trimethylamine are detected in the exhaled breath of uremic patients (Fig. 2c2)^59–61^. There are two different modes of ammonia metabolism in the human body: the urea cycle and hemodialysis. The detailed process is presented in Fig. 2d. Endogenous ammonia is a product of protein metabolism and is converted to urea in the liver and subsequently eliminated by the glomerulus (urea cycle); this results in its depletion in the exhaled breath of the H subjects. However, in patients with impaired renal function, the proportion of ammonia in the exhaled breath is elevated, indicating an altered exhaled breath profile. Remarkably, hemodialysis treatment has been found to effectively reduce the level of ammonia^7^.

Sulfur compounds found within the human body are derived from the incomplete metabolism of methionine through the transamination pathway. They serve as the main markers for liver failure^62^. Remarkably, patients who have undergone liver transplantation or are affected by liver disease exhibit comparatively high concentrations of sulfur compounds in their exhaled breath. Specifically, the exhaled breath of individuals with liver disease shows significant increases in the levels of dimethyl sulfide, acetone, 2-butanone and 2-pentanone^63^. Importantly, liver disease is an important extraoral cause of halitosis^62^. In fact, approximately 85% of halitosis cases stem from lesions located within the oropharynx, such as tongue coating, gingivitis, periodontitis, and tonsillitis. These conditions are associated with sulfur-containing compounds, such as hydrogen sulfide, methyl mercaptan, and dimethyl sulfide^64,65^.

## Exhaled breath analysis technology

Exhaled breath analysis in academic research entails the utilization of several prevalent techniques. Notably, gas chromatography (GC) and mass spectrometry (MS) are extensively used, relying on substantial analytical instruments. Another prevalent approach is cavity ring-down spectroscopy (CRDS) based on spectral analysis. Additionally, gas sensor analysis grounded in electrochemical principles constitutes a significant methodological avenue^18,66^. Herein, a concise summary of the detection methods and underlying principles specific to each technique is provided below.

**Fig. 3:**
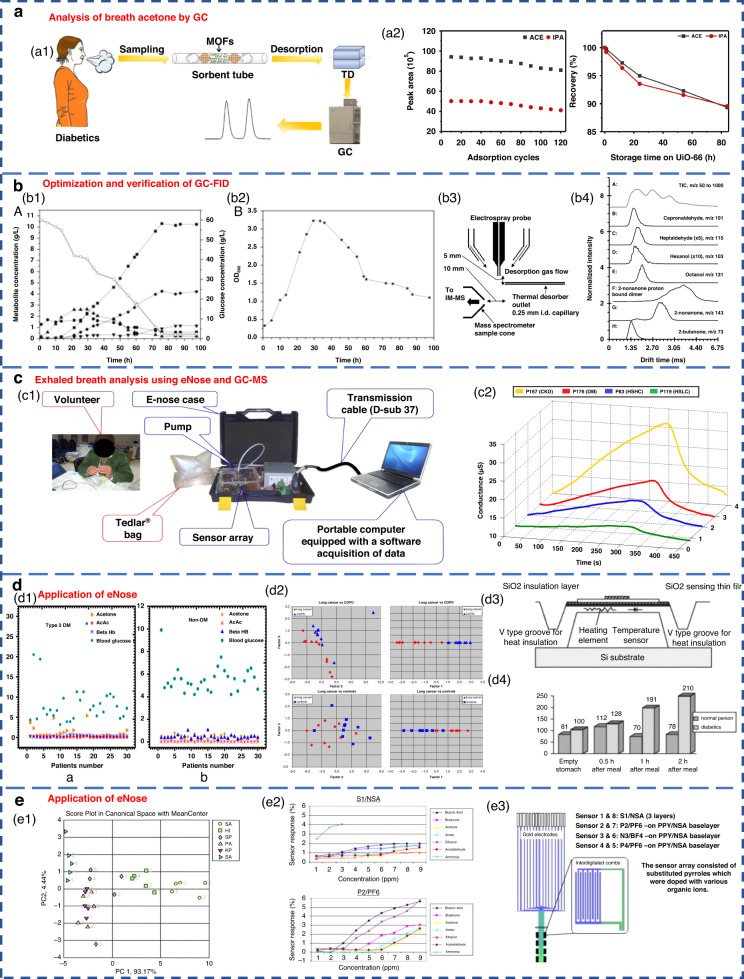
Detection of human exhalation based on different analysis techniques. **a** Acetone content in the breath of diabetic patients analyzed by GC^68^. Copyright 2019 Springer Nature. **b** Optimization and verification of the GC-FID determination method. **b1**, **b2** Concentration distribution of ABE fermentation substrate and product^50^. Copyright 2014 Oxford University Press. **b3**, **b4** Detection of VOCs in breath using thermal desorption electrospray ionization-IMS-MS^73^. Copyright 2021 American Chemical Society. **c** Exhaled breath analysis using eNose and GC-MS^63^. Copyright 2018 Elsevier. **c1** Experimental setup of the eNose system. **c2** Electrical conductance changes in the presence of 4 VOC samples using the MQ-137 sensor. **d** Application of eNose in the exhaled breath of diabetic, NSCLC and COPD patients. **d1** Scatter plot for plasma breath acetone in type 2 diabetic (left) and nondiabetic mellitus patients (right)^23^. Copyright 2019 MDPI. **d2** eNose results for the discrimination of patients with NSCLC and COPD^75^. Copyright 2009 Elsevier. **d3**, **d4** Novel method for diabetes diagnosis based on eNose^77^. Copyright 1997 Elsevier. **e** Application of eNose in upper respiratory tract infection and wound bacteria detection. **e1** Identification of upper respiratory bacterial pathogens with eNose^24^. Copyright 2009 John Wiley & Sons. **e2**, **e3** Development of CP sensor arrays for wound monitoring^25^. Copyright 2008 Elsevier

GC separates various components based on their differential distribution coefficients in the relative motion of two phases. In terms of reliability, GC is recognized as the best standard solution for gas detection^67^. The acetone content in the breath of diabetic patients can be effectively analyzed by GC (Fig. 3a). Currently, gas detection methods utilizing GC primarily include thermal desorption-gas chromatography (TD-GC)^68^, gas chromatography-hydrogen flame ionization detector (GC-FID)^50^, gas chromatography-ion mobility spectrometry (GC-IMS)^69^ and gas chromatography-mass spectrometry (GC-MS)^62^. The distribution of the acetone-butanol-ethanol (ABE) fermentation substrate was tested based on the GC-FID method, as well as the product concentration (Fig. 3b1-2). However, due to the limited qualitative capacity of GC, it was imperative to combine GC with other detectors for more precise analysis. Furthermore, GC exhibits drawbacks, such as lengthy detection times, complex operational mechanisms, and the requirement for skilled personnel, causing it to be less suitable for point-of-care testing in medical diagnostics^70^.

MS entails the ionization of gases into charged particles via an ion source, followed by their separation based on the mass-to-charge ratio utilizing electric and magnetic fields. It has the advantages of fast response and no pretreatment. At present, proton transfer reaction-mass spectrometry (PTR-MS)^71^, selective ion flow tube-mass spectrometry (SIFT-MS)^72^ and IMS-MS^73^ are widely employed for exhaled gas analysis. Thermal desorption electrospray ionization-IMS-MS can also be used to detect VOCs in breath (Fig. 3b3-4). Tarik’s research team performed noninvasive diagnosis of chronic kidney disease, diabetes, and H subjects using eNose and GC-MS coupled analysis^63^. Breath samples were measured with an eNose system specifically developed for breath analysis purposes (Fig. 3c1). Typical responses produced by the MQ-137 sensor in the presence of different breath samples (chronic kidney disease, diabetics and H subjects with high/low creatinine) are shown in Fig. 3c2. However, many kinds of trace gases are present in human exhaled breath, which leads to the inevitable formation of numerous ionic clusters. Consequently, when there were components with identical mass-to-charge ratios in exhaled breath samples, clearly distinguishing these components by MS alone was difficult. In addition, MS has a great requirement of a high vacuum level within the test chamber. Therefore, the equipment structure is complex, limiting the development of portability and miniaturization.

CRDS stands out for its remarkable sensitivity. It is widely used in the trace detection of gases as well as absorption spectroscopy of molecules, atoms and clusters. Wang et al. from Mississippi State University first used CRDS technology to systematically study acetone in human exhaled gas and its correlation with blood glucose concentration in 2010^74^. CRDS leverages gas-specific optical absorption peaks to detect trace gases. Moreover, it is not affected by the laser intensity fluctuation. However, its utilization is constrained by the availability of laser light sources and high reflectivity mirrors. Acquiring CRDS instruments for multiple wavelength ranges can be challenging. Additionally, the equipment needs to be highly calibrated and is expensive.

The abovementioned three methods have high requirements for experimental instruments and environmental conditions. Typically, the detection and analysis processes take a long time and cannot be monitored in real time. Additionally, the equipment structures are complex, impeding progress in terms of portability and miniaturization. Furthermore, the large cost associated with these methods hinders their widespread adoption and development across various fields.

Compared with the above methods, the gas sensor analysis method can quickly obtain qualitative and quantitative gas detection results. They provide high sensitivity, small size, ease of packaging, and low price. By using a sensor array comprising multiple sensors, collaborative analysis of gas samples can also be achieved. Based on the principles of biological olfaction, eNose technology utilizes gas sensor arrays and PRA for gas detection and has shown excellent performance and significant application potential. Notably, it has been applied in clinical medicine, including early screening of diverse cancers^75^, lung diseases^76^, diabetes^77^, bacterial pathogen identification^78,79^, and in the analysis of microbial metabolites from superficial wounds^25,80^.

Respiratory acetone levels were investigated in diabetic and nondiabetic patients by using an eNose system (Fig. 3d1, 3-4). As expected, diabetic patients exhibited high levels of respiratory acetone (greater than 0.8 ppm) compared to their nondiabetic counterparts (less than 0.8 ppm). The applications of eNose in distinguishing non-small cell lung cancer (NSCLC) and COPD patients are shown in Fig. 3d2. The eNose system was able to distinguish the LC patients from the COPD patients and H subjects from the breath test experimental results. This result confirmed that eNose has the potential to become a noninvasive diagnostic tool for LC patients in the future. Recent studies have demonstrated the ability of eNose technology to test for bacterial infections (Fig. 3e1). The eNose analysis exhibited the ability not only to detect common upper respiratory pathogens but also to discriminate between bacterial species when compared to the control group. Moreover, eNose sensor arrays based on conductive polymers can also be used for wound monitoring (Fig. 3e2-3).

Breath analysis technology based on eNose possesses the advantages of high sensitivity, rapid response, real-time monitoring, and user-friendly portability. As a noninvasive diagnostic model, it presents an ideal approach for the rapid screening of diseases through breath detection. The eNose system consists of two key technologies: the sensor array responsible for the detection of chemical substances and the algorithm for providing the analytical software model within the system.

## Gas sensors for eNose systems

**Fig. 4:**
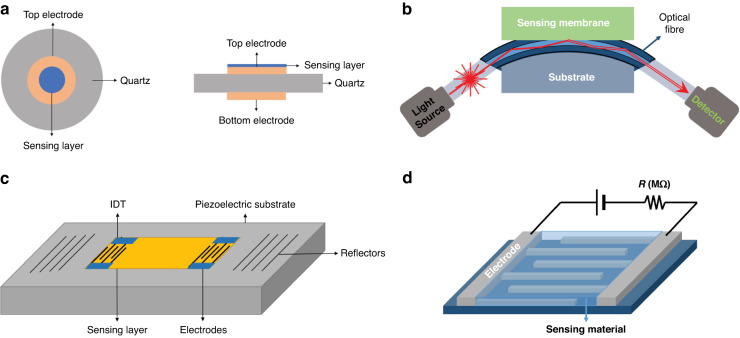
Typical sensors for eNose technology. Schematic view of a typical QCM sensor (**a**), fiber-optic sensor (**b**), SAW gas sensor (**c**), and chemical resistance gas sensor (**d**)^129^. Copyright 2019 MDPI

eNose technology relies on gas sensors to obtain the composition information of gas samples. To enable precise detection of breath-related diseases with complex components, integration of multiple specific sensors into a sensing array is needed to achieve high-precision detection^81^. In the field of exhalation analysis, sensor arrays have been recognized for their considerable application potential^82^. In the field of clinical practice, several types of gas sensors find widespread utilization in eNose systems. These include the following: chemical resistance sensors, such as metal oxide semiconductor (MOS) sensors and conductive polymer (CP) sensors; the widely used piezoelectric sensors, such as quartz crystal microbalance (QCM) sensors and surface acoustic wave (SAW) sensors; electrochemical (EC) sensors; and optical sensors^19,70,82–85^. Typical schematics are shown in Fig. 4.

### Chemical resistance gas sensor

The MOS sensor, a member of the chemical resistance gas sensor category, is the most commonly used sensor type used in eNose systems^7,81^. It has the advantages of high sensitivity, rapid response, miniaturization, low cost, user-friendliness, and good compatibility with microelectronic processes^18^. MOS sensors operate by utilizing the adsorption of the targeted gas to modify the conductivity of the semiconductor material. According to the difference in charge carriers, they can be divided into N-type and P-type semiconductor materials. Notably, these two semiconductor materials have different sensing responses to reducing gas and oxidizing gas, as shown in Table 2.

**Table 2:** Sensing response of n-type and p-type semiconductor materials to reducing gas and oxidizing gas

	n-type	p-type	Gas	Ref.
Charge carrier	Electron	Hole	/	
Reducing gas	Resistance reduction	Resistance rise	Ethanol, acetone, NH_3_, H_2_S, CO,	^81,169^
Combustion gas	Resistance rise	Resistance reduction	NO_2_, O_3_	

At present, a range of MOS sensing materials, such as SnO_2_, ZnO, CuO, TiO_2_, WO_3_, NiO, In_2_O_3_, WO_3_, TiO_2_, Fe_2_O_3_, and MoO_3_, are commonly used to detect various gases, such as acetone, ethanol, formaldehyde, H_2_S, NH_3_, NO_2_, and CO^19,28,82,83^. The performance of the MOS sensor is influenced by the morphology of the sensing material as well as surface additives. Semiconductor materials are generally polycrystalline materials containing lattice gaps between the crystalline structures. During the charge transport process, the grain boundary barrier affects the material resistance to a certain extent. Therefore, the selectivity and sensitivity to the target gas can be increased by increasing the porosity or reducing the grain size to the nanoscale level; these methods expand the specific surface area and generates oxygen-rich vacancies^2,18,83^. The sensitivity can be defined as R_a_/R_g_ (for reducing gases) or R_g_/R_a_ (for oxidizing gases), where R_a_ represents the resistance of the gas sensor in the reference gas (generally air) and R_g_ represents the resistance of the gas sensor in the reference gas containing the target gas^86^.

**Fig. 5:**
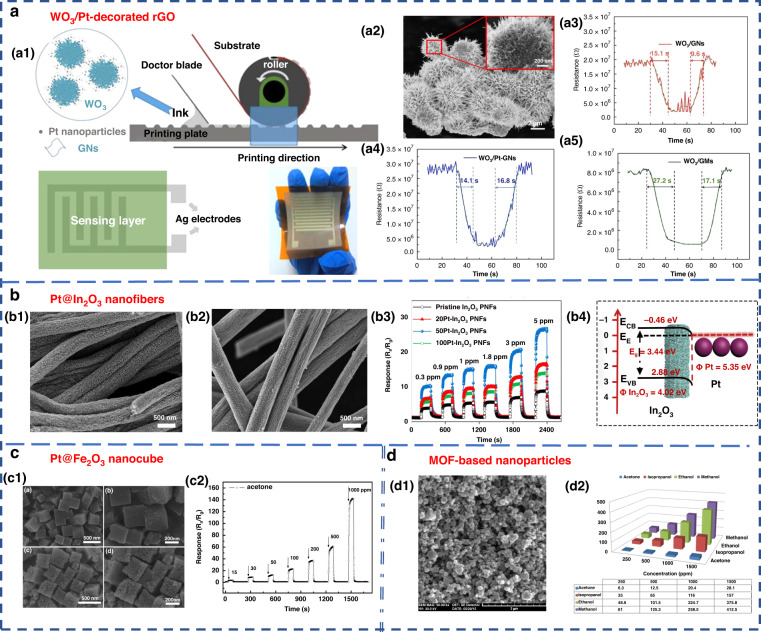
Common MOS sensing materials and sensing mechanism. **a** WO_3_/Pt-decorated rGO nanosheets for the detection of acetone^88^. **a1** Schematic representation of the gravure printing process. **a2** SEM image. **a3**–**a5** Response transient of the gas sensor based on WO_3_/GNs, WO_3_/Pt-GNs and WO_3_/GMs samples to 10 ppm acetone at 200 °C. Copyright 2017 Elsevier. **b** In_2_O_3_ nanofiber-functionalized Pt catalysts^92^. **b1**, **b2** SEM images. **b3** Linear relationship between response and acetone concentrations. **b4** Schematic illustration of the energy band of Pt-In_2_O_3_ PNFs. Copyright 2019 Elsevier. **c** Acetone gas sensor based on nanosized Pt-loaded Fe_2_O_3_ nanocubes^93^. **c1** SEM images of pure Fe_2_O_3_ (top) and Pt-Fe_2_O_3_ (bottom). **c2** Response curve of Pt-Fe_2_O_3_ exposed to a high concentration of acetone at 139 °C. Copyright 2019 Elsevier. **d** MOF-based nanoparticles for VOC detection^89^. **d1** SEM image. **d2** Sensor sensitivity for methanol, ethanol, isopropanol and acetone at different concentrations of 250, 500, 1000 and 1500 ppm. Copyright 2016 Elsevier

Nanostructured materials, such as nanowires, nanosheets, nanospheres, and nanopetals, have been used for VOC detection^87–90,91^. Additionally, modifying the surface of the material by adding a certain number of additives is another way to enhance the performance of MOS sensors and improve their selectivity, sensitivity and response speed^2,83^. Examples of such additives include Pt-In_2_O_3_, Pt-Fe_2_O_3_, Co-SnO_2_, Au-ZnO, Si-WO_3_^92–96^, and composite metal oxides, such as La_2_O_3_-SnO_2_, In-WO_3_-SnO_2_, and ZnO-SnO_2_^97–99^. Chen et al. designed and developed gravure-printed WO_3_/Pt-modified rGO (reduced Graphene Oxide) nanosheets for the detection of acetone^88^. As shown in Fig. 5a, the transient response to 10 ppm acetone was shown for three different samples and provided response/recovery times of approximately 15.2/9.6 s and 14.1/6.8 s for WO_3_/GNs and WO_3_/Pt-GNs, respectively. Notably, the gas response/recovery times were much lower than those of WO_3_/GMs. The fast response recovery characteristics were attributed to the large number of p-n junction active sites present at the WO_3_/rGO interface, which facilitated the rapid charge carrier transport into the conduction band. Liu’s group designed an acetone gas sensor based on a porous platinum (Pt)-doped In_2_O_3_ nanofiber structure (Fig. 5b)^92^. Similar work was performed by Zhang’s group to design and fabricate an acetone sensor based on nanosized Pt-loaded Fe_2_O_3_ nanocubes (Fig. 5c)^93^. Additionally, Homayoonnia et al. developed metal-organic framework (MOF)-based nanoparticles for VOC detection (Fig. 5d)^89^.

CP sensors are also chemical resistance sensors^100^ that provide high sensitivity, high selectivity and the ability to function at room temperature^19^. The material properties of CP are similar to those of some metal and inorganic semiconductor materials, while retaining the flexibility of the polymer and having the advantage of easy processing and synthesis^101^. Common examples of CPs include polypyrrole (PPy), polyaniline (PANI), and polythiophene (PT)^102–104^. Researchers have explored the potential of CP sensors within eNose for detecting VOCs. Chatterjee et al. developed an eNose system by integrating 5 carbon nanotube (CNT)-based CP nanocomposite (CPC) sensors with a CNT sensor^105^. The system was able to successfully detect 18 different LC VOC biomarkers at the ppm level; thus, its application performance was confirmed. João et al. used the commercial Cyranose 320 (Sensigent, Baldwin Park, CA, USA) eNose device to effectively distinguish asthma patients through the analysis of their breath VOCs. The device utilized a NoseChip nanocomposite array consisting of 32 CP sensors. The sensor consisted of a carbon black film dispersed in a polymer matrix, which was deposited onto two metal electrodes to form an electrical connection. The relative resistance change of sensors was measured upon exposure to VOCs^106^. Finnegan et al. proposed a miniature, low-cost, and battery-free wearable eNose based on a CP sensor array^107^. This device could be used to detect 6 VOCs: pyridine, tetrahydrofuran, ethanol, methanol, acetic acid and ammonium hydroxide^107^.

### Piezoelectric gas sensor

SAW and QCM sensors are two widely used piezoelectric sensors in eNose applications^19^. SAW sensors use the mutual conversion of electrical energy and mechanical energy to generate sound waves through piezoelectric materials. When sound waves propagate through the piezoelectric substrate or on the surface of the piezoelectric substrate, any change in the propagation path characteristics leads to changes in the SAW characteristics, which can be associated with the measured physical (or chemical) quantities^82^. SAW technology has evident advantages of high sensitivity and low energy consumption. However, the process of manufacturing patterned metal electrodes on piezoelectric substrates is expensive and complex, requiring specialized equipment. Additionally, it is very sensitive to environmental factors, such as temperature and humidity, limiting its application^85,108^.

**Fig. 6:**
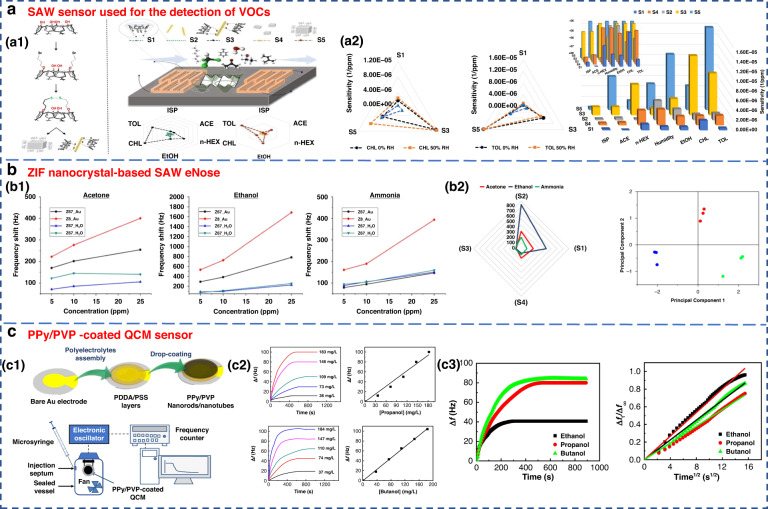
Examples of SAW and QCM sensors. **a** SAW sensor used for the detection of VOCs^182^. **a1** Surface reaction mechanism diagram of the SAW sensor. **a2** Sensitivity of sensors S1–S5 to 6 gases under 50% relative humidity (RH). Copyright 2021 Elsevier. **b** ZIF nanocrystal-based SAW eNose to detect diabetes in human breath^109^. **b1** Calibration curves of sensors S1, S2, S3 and S4 for acetone (left), ethanol (middle) and ammonia (right). **b2** Radial representation of the sensor array responses to 10 ppm of acetone, ethanol and ammonia. Copyright 2018 MDPI. **c** Gas sensing properties of a PPy/PVP nanorod/nanotube-coated QCM sensor^171^. **c1** Illustration of PPy/PVP nanorod/nanotube film formation. **c2** Time-dependent frequency change of the QCM sensor when exposed to different concentrations of 1-propanol and 1-butanol and their calibration curves. **c3** Frequency change of the QCM sensor against exposure time for a constant concentration of ethanol, 1-propanol and 1-butanol vapor (184 mg L^−1^) and plot of Δft/Δf∞ against the square root of time. Copyright 2021 Elsevier

FundaKus et al. studied the molecular recognition properties of Calix arene-modified gold nanorods (AuNR) and silver nanoclusters (AgNC) on the surface of SAW transducers (Fig. 6a)^4^. The sensitivity of the modified sensor was 6–8 times higher when used to detect acetone, ethanol, chloroform, n-hexane, toluene and isoprene. The use of zeolitic imidazolate framework (ZIF) nanocrystals as a sensitive layer in SAW-based sensor arrays was developed by Fabio et al. As shown in Fig. 6b, it could detect and identify three diabetes-related breath markers of acetone, ethanol and ammonia with a detection limit of 5 ppm^109^.

QCM is a type of bulk acoustic wave (BAW) device made of quartz, which is mainly cut by AT^110^. It has received considerable attention due to its high precision and sensitivity^111,112^. As a piezoelectric mass sensor, QCM measures changes in the resonance frequency when specific gas molecules are adsorbed on the sensing material’s surface. By measuring the change in resonance frequency, the mass or concentration of a specific gas adsorbed can be quantified^70,81^. The sensing performance of QCM depends on the physical or chemical properties of coating materials, such as zeolites, CNTs and polymers, which have been used to detect gases on the surface of QCM^82^. A QCM sensor coated with a colloidal PPy/poly(N-vinylpyrrolidone) (PPy/PVP) nanorod/nanotube film was used for the detection of alcohol vapors (Fig. 6c). This sensor showed good detection sensitivity for alcohol vapor.

### Electrochemical sensor

**Fig. 7:**
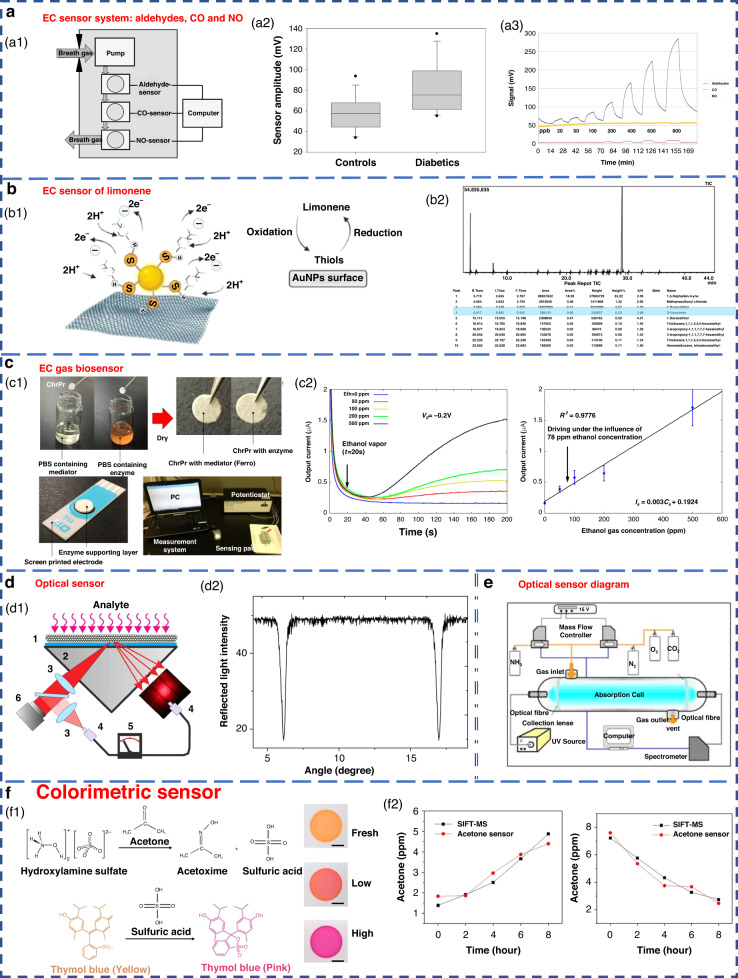
Examples of electronic and optical sensors. **a** EC sensor system for breath analysis of aldehydes, CO and NO^171^. **a1** Schematic of the sensor system. **a2** Comparison of the aldehyde signals from the breath of controls and diabetic patients. **a3** Response of the sensor system to dry aldehyde standards (20–800 ppbV) in clean ambient air. Copyright 2015 IOP Publishing. **b** EC sensor of limonene using thiol-capped gold nanoparticles^117^. **b1** Schematic diagram of limonene oxidation at the electrode surface. **b2** Screening of limonene via GC-MS. Copyright 2022 Elsevier. **c** An EC gas biosensor based on enzymes immobilized on chromatography paper^120^. **c1** Synthesis of the sensitive materials and flow chart of the sensor fabrication. **c2** Typical current responses of modified chromatography paper enzyme electrodes for several ethanol gaseous concentrations. Copyright 2017 MDPI. **d** Optical sensors with high sensitivity and fast response^122^. **d1** Schematic of the experimental setup. **d2** Measured angular dependence of the reflected light intensity. Copyright 2015 Elsevier. **e** Experimental device diagram of ammonia sensing using an optical sensor^183^. Copyright 2009 Elsevier. **f** Colorimetric sensor for detecting exhaled acetone^127^. Copyright 2021 American Chemical Society

The EC sensor operates by analyzing the concentration of the gas being measured. It detects changes in the current generated by the oxidation or reduction reaction of gas molecules on the surface of the catalytic electrode. This type of sensor is particularly effective in detecting electrochemically active gases^113,114^. However, it has a lower sensitivity to a variety of compounds, especially aromatic hydrocarbons^115^. Obermeier et al. developed an eNose system composed of three different EC sensors. As shown in Fig. 7a, it could be used to detect ppb levels of exhaled aldehydes and airway inflammation markers, such as CO and NO^116^. The Nazir group developed a hexanol-terminated AuNP-based eNose system for detecting limonene (Fig. 7b), a biomarker of exhaled breath found in patients with cirrhosis. The detection results of this system provided an R^2^ value of 0.99. The qualitative and quantitative detection results were close to those of GC-MS^117^.

Some EC sensors for breath gas detection are enzyme sensors^118–121^. Due to the specific reactivity of enzymes, they have high sensitivity and high selectivity. However, an enzyme is sensitive to temperature and needs to be stored at low temperature. Furthermore, the enzyme sensor is disposable and cannot be repeatedly tested^83^. An EC gas biosensor based on an enzyme immobilized on chromatographic paper is shown in Fig. 7c. Ethanol vapor could be measure in the concentration range of 50–500 ppm.

### Optical gas sensor

Optical sensors have the advantages of high sensitivity, good selectivity, and rapid response. They also have the ability to monitor chemical and physical parameters on a large scale^122–124^. These sensors can operate in colorimetric, fluorescence, chemiluminescence or scattering modes, converting the optical changes generated by the interaction between the analyte and the biometric substance into measurable signals^49,82^.

In recent years, there have been highly sensitive fast response gas sensors based on light reflection at the glass-photonic crystal interface (Fig. 7d), which have a sensitivity of 1 ppm for NH_3_, a rise time response of 100 ms, and a recovery time of approximately 10 s. A schematic diagram of the optical sensor ammonia sensing experimental setup is shown in Fig. 7e. However, the optical sensor equipment system is complex and costly to operate^125^. Additionally, the optical system results can be easily affected by external factors, such as physical damage and sunlight; this greatly limits its miniaturization and portability^49,100^.

Colorimetric sensors are optical sensors that produce visible visual color changes when affected by external stimuli. Gold, silver, copper and other nanoparticles are widely used in colorimetric sensing because of their favorable optical properties^83^. Colorimetric acetone sensors have shown promising application potential in detecting human exhaled VOCs due to their advantages of simple production and rapid detection capabilities (Fig. 7f)^126,127^.

### Summary of this chapter

From the perspective of medical diagnosis, the ideal sensor array in eNose should have the advantages of high sensitivity, stable performance, rapid response, simple portability, reusability and low cost^19,83^. The results of the relevant studies are summarized in terms of chemical resistance gas sensors, piezoelectric gas sensors and electrochemical sensors in Table 3. Relevant target analytes, practical detection ranges and detection limits are also detailed.

**Table 3:** Main features of various eNose sensors

Sensor type	Working principle	Advantage/Disadvantage	Target detector	Sensitivity	Detection Range/Limit	Ref.
Chemical resistance gas sensor	Resistance change	Low cost, easy to use, fast response speed/high test temperature, poor selectivity	Acetone	24.9@50 ppm	0.5–100 ppm 103 ppb	^94^
			Ethanol	6.76@50 ppm	1–50 ppm 90 ppb	^170^
			Methylbenzene	>10@2.5 ppm	–	^105^
Piezoelectric gas sensor	Resonance frequency change	High sensitivity/hard to implement, poor signal-to-noise ratio	RH	29.0 Hz/%RH	0–97%	^111^
			1*-*butanol	0.5709 Hz mg/L	35.7–184 mg/L 9.48 mg/L	^171^
EC sensor	Gas reaction produces ion movement	High sensitivity, low power consumption/short life, integrated packaging difficult	Nitrite	–	0.5–50 μg/mL 4 μmol/L	^113^
			Carbon dioxide	~0.132 mV/ppm	160–2677 ppm	^118^

## Pattern recognition algorithm used within the eNose system

Pattern recognition refers to identifying trends or specific patterns in data^81^. The core processing technology in the eNose system involves the qualitative or quantitative analysis of gas information obtained by a sensor array through a machine learning algorithm^85,128^. However, in real-world disease breath diagnosis, the eNose system must deal with a diverse array of complex and trace gases. To address this challenge, researchers have incorporated appropriate multivariate analysis technology into the algorithm components of the eNose system, resulting in improved selectivity in multivariate scenarios. This approach effectively mitigates the problem of low cross-sensitivity and poor selectivity observed in existing gas sensors^19^. In addition, for various diseases, the detection limits of the corresponding markers are different (Table 1). A single sensor has difficulty meeting the detection limits of different markers alone, and the use of a sensor array of the eNose system effectively solves this problem. Then, the gas information obtained by the sensor array is qualitatively or quantitatively analyzed by a machine learning algorithm to meet the practical application of the eNose system in the field of human breath. The practical application of PRA in assisting eNose for disease breath diagnosis in recent years is generalized in Table 4. Abbreviations in Table 4 are summarized in Table 5.

**Table 4:** Application of eNose technology in exhalation diagnosis of diseases

Disease	Gas detection device^a^	PRA	Sample status	Result	Ref
LC	Cyranose 320	LRA	Nonsmoking: PG *n* = 133; HG *n* = 132; Smoking: PG *n* = 119; HG *n* = 91	Nonsmoking: Se = 96.2%; Sp = 90.6%; Smoking: Se = 95.8%; Sp = 92.3%	^172^
LC	Aeonose;	ANN	PG *n* = 52; HG *n* = 93	Se = 83%; Sp = 84%	^173^
LC	TGS2600/2602/822; MQ3	ANN	PG *n* = 6; HG *n* = 10	A = 93.8%; Se = 85.7%; Sp = 100%	^174^
COPD	FGC eNose	PCA	PG *n* = 23; HG *n* = 33	A = 82.2%; Se = 96%; Sp = 91%	^175^
Asthma	Cyranose 320	PCA combined with penalized LRA	Asymptomatic: CG *n* = 10; Controllable *n* = 9 Symptomatic: Partially controlled *n* = 7. Uncontrolled *n* = 12	Se = 79%; Sp = 84%	^176^
BO	Aeonose	ANN	PG *n* = 129; GRP *n* = 141; CG *n* = 132	Se = 91%; Sp = 74%	^177^
CRC	Aeonose	ANN	CRC *n* = 70; AAs *n* = 117; Non-AAS *n* = 117; HPs *n* = 15; Colonoscopy normal *n* = 128	CRC: AUC = 0.84; Se = 95%; Sp = 64%. AAs: AUC = 0.73; Se = 79%; Sp = 59%	^178^
ILD	SpiroNose	PLS-DA	Sarcoidosis *n* = 141; IPF *n* = 85; ILD *n* = 33; CAP *n* = 25; INIP *n* = 10; IPAC *n* = 11; Other ILD *n* = 17; CG *n* = 48	ILD and Control group: T/V set AUC = 1/1; IPF and other ILD patients: T/V set AUC = 0.91/0.87; Individual diseases: 0.85 < AUC < 0.99	^179^
COVID-19	Gold nanoparticles (8) sensor array	QDA; LDA; ROC curve analysis	COVID-19 PG *n* = 49; NCPIG *n* = 33; HG *n* = 58	Patients and HG: T/T set A = 94%/76%; COVID-19 and NCPIG: T/T set A = 90%/95%	^180^
LC, COPD	TGS2600/2610/2620/822/826	SVM	LC *n* = 27; COPD *n* = 22; HG *n* = 39	LC: A = 88.79%; Se = 89.58%; Sp = 88.23%. COPD: A = 78.70%; Se = 72.50%; Sp = 82.35%	^181^

^a^Cyranose 320 (Smith’s Detection, Pasadena, CA, USA); Aeonose (the eNose Company, Zutphen, the Netherlands); TGS2600, TGS2610, TGS2620, TGS2602, TGS822, TGS826 (Figaro, USA); MQ3 (Parallax, USA); FGC eNose (HERACLES II, Alpha MOS Company, Toulouse, France); SpiroNose (Breathomix, Leiden, The Netherlands)

**Table 5:** List of abbreviations (in alphabetic order)

	Abbreviation	Full-title
A	A	Accuracy
	AAS	Advanced adenomas
	ANN	Artificial neural network
	AUC	The area under the receiver operating curve
B	BO	Barrett’s esophagus
C	CAP	Chronic allergic pneumonia
	CG	Control group
	COPD	Chronic obstructive pulmonary disease
	COVID-19	Corona Virus Disease 2019
	CRC	Colorectal cancer
	CRF	Chronic renal failure
G	GRP	Gastroesophageal reflux patients
H	HG	Healthy group
	HPs	Hyperplastic polyps
I	ILD	Interstitial lung disease
	INIP	Idiopathic nonspecific interstitial pneumonia
	IPAC	Interstitial pneumonia with autoimmune characteristics
	IPF	Idiopathic pulmonary fibrosis
L	LC	Lung cancer
	LRA	Logistic regression analysis
N	NCPIG	Non-COVID pulmonary infection control group
P	PCA	Principal component analysis
	PG	Patients’ group
	PLS-DA	Partial least squares discriminant analysis
Q	QDA	Quadratic discriminant analysis
R	ROC	Receiver operating characteristic
S	Se	Sensitivity
	Sp	Specificity
	SVM	Support vector machine
T	T/T	Training/test
	T/V	Training/validation

Gas sensor arrays in the eNose system are typically analyzed using classical machine learning algorithms, such as principal component analysis (PCA)^6,82,115,129,130^, linear discriminant analysis (LDA)^6,19,82,130,131^, support vector machine (SVM)^2,6,19,70,130,132,133^, decision tree (DT)^2,130^, K-nearest neighbor (KNN)^2,6,19,130,134^, cluster analysis (CA)^115^, canonical discriminant analysis (CDA)^115^, partial least squares regression (PLS)^63^, and others.

**Fig. 8:**
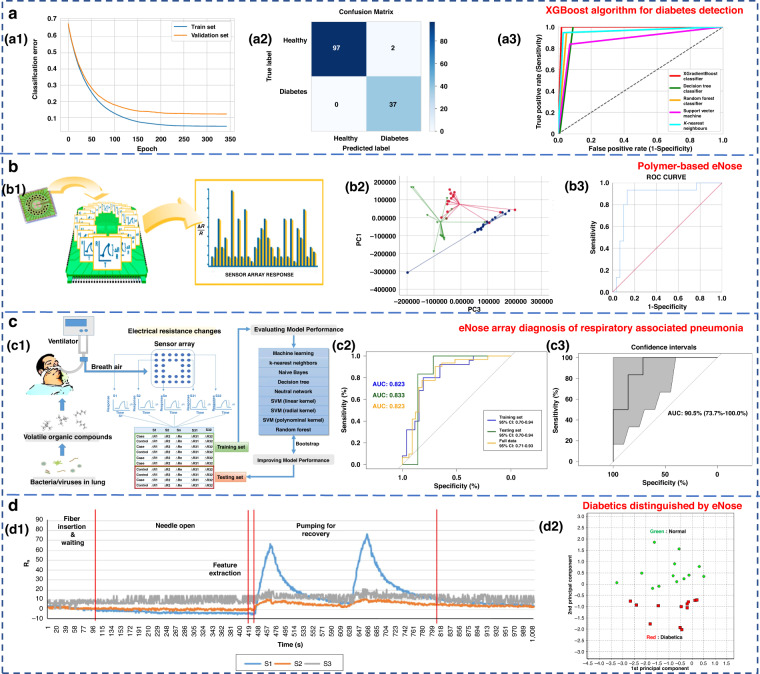
Examples of pattern recognition algorithms for eNose systems. **a** Artificial breath classification using the XGBoost algorithm for diabetes detection^136^. **a1** XGBoost learning curves. **a2** XGBoost classifier confusion matrix. **a3** ROC comparison of different algorithms. Copyright 2021 MDPI. **b** Role of polymer-based eNose in the detection of head and neck cancer from exhaled breath^137^. **b1** Working principal scheme of Cyranose 320. **b2** Two-dimensional PCA with 2 composite factors. **b3** ROC curve with line of identity of the breath print discriminant function (representing PC1 and PC3). Copyright 2022 MDPI. **c** eNose sensor array signal diagnosis of respiratory-associated pneumonia^141^. **c1** Flow diagram of this study. **c2** Area under the receiver operating curve (AUC) for VAP in the training set, testing set, and full dataset. **c3** AUC for VAP in the testing set, with the 95% confidence interval. Copyright 2020 Springer Nature. **d** Diabetics distinguished by using eNose^144^. **d1** Sensor response of breath samples of the control group. **d2** PCA result of measured breath samples. Copyright 2018 John Wiley & Sons

Ensemble learning is a machine learning strategy independent of the algorithm^135^. It can combine a group of weak learners to form a strong one. The generation method of the learner can be roughly divided into two categories: Boosting, in which there is a strong dependence between individual learners and serial generation can only be used; and bagging, in which there is no strong dependence between individual learners, and parallel generation can be used. Paleczek et al. proposed a diabetic breath detection method based on the XGBoost algorithm (Fig. 8a). The system had high selectivity for low concentrations of acetone. Its accuracy and recall rates were 99% and 100%, respectively, which were superior to those of other commonly used algorithms (such as SVM, KNN and DT)^136^.

To investigate the potential of eNose in detecting head and neck cancer through exhaled breath analysis, Roberta’s research team used Cyranose 320 for sampling, as depicted in Fig. 8b^137^. In the PCA diagram, patients with head and neck cancer formed distinct clusters in relation to both the control group and patients with allergic rhinitis. The three groups were successfully discriminated with a typical discriminant analysis, and a cross-validation accuracy of 75.1% (*p* < 0.01) was achieved. The area under the receiver operating characteristic (ROC) curve for identifying patients with head and neck tumors from other groups reached 0.87. In conclusion, eNose technology exhibits promising application potential in diagnostic contexts. Lei et al. proposed a high-precision PCA-SVE ensemble learning framework that combined 11 four-type gas sensors to form an eNose system for rapid noninvasive exhalation diagnosis of LC^135^. A set of single machine learning models with excellent performance, including SVM, DT, random forest (RF), logistic regression and KNN, were selected to construct the PCA-SVE framework. Experiments were performed on 214 exhaled breath samples (98 LC patients and 116 H subjects). The accuracy, sensitivity and specificity of the proposed framework were 95.75%, 94.78% and 96.96%, respectively.

Due to their strong self-learning and adaptive ability, as well as nonlinear expression ability, neural networks often have better analysis results than traditional machine learning methods when dealing with complex and trace human exhaled breath data. The commonly used neural networks in the eNose systems are artificial neural networks (ANNs)^115^, multilayer perceptron neural networks (MLPs)^138^, convolutional neural networks (CNNs)^138–140^, and radial basis functions (RBFs)^115^. Chen et al. diagnosed ventilator-associated pneumonia (VAP) by sensor arrays and machine learning technology (Fig. 8c)^141^. Eight algorithms, including KNN, naive Bayes, DT, neural network, SVM (including linear kernel, polynomial kernel and radial basis kernel), and RF, were used. The results were verified by using real exhaled samples from VAP patients (*n* = 33) and a control group (*n* = 26), with an average accuracy of 0.81 ± 0.04, a sensitivity of 0.79 ± 0.08, and a specificity of 0.83 ± 0.00^136^. Hendrick et al. identified tuberculosis by using a sensor array combined with a pattern recognition method. The classification effects of SVM, XGBoost, ANN and RF were researched. The accuracy rates were 92%, 88.24%, 94.87% and 84.24%, respectively^142,143^.

Jin et al. selected four kinds of semiconductor chemical sensors with different sensitive materials (Au/N-SnO_2_, Au/N-WO_3_, N-WO_3_ and N-SnO_2_) and constructed a 20-sensor array operating at five different temperatures (245, 285, 310, 325, and 340 °C)^144^. The work is shown in Fig. 8d. PCA and Euclidean distance were used to identify the best-performing sensor array combination and enabled the accurate detection of five types of VOC gases, including acetone. Twenty-five real exhalation samples (12 diabetic patients and 13 H subjects) were successfully distinguished. Although classical machine learning methods are simple to design and have a relatively fixed framework with few parameters, their generalization ability is weak. Consequently, it is difficult to accurately identify the gas atmosphere in high-noise environments, such as exhaled breath detection.

By imitating the cognitive process of the human brain, the neural network achieves high-precision recognition and analysis of the target by designing parameters, such as the number of network layers, the number of neurons, and the activation functions. Typically, the performance of neural networks improves with an increase in the number of data samples acquired^130^.

## Development of the eNose system

eNose has a documented history dating back to 1964^145^, when Wilkens and Hartman used electrodes to chemically react with gases to simulate the olfactory process of organisms. Since then, a large number of experts and scholars have been attracted to this field and carried out research.

A significant breakthrough in eNose research occurred during the annual meeting of the European Chemical Sensing Research Organization held at the University of Warwick, England in 1987^146^. At this meeting, researchers from the University of Warwick presented a paper on gas sensors that introduced the concept of ‘pattern recognition’ and discussed the feasibility of using sensors for detecting both composite and simple gases. Following several years of exploration in eNose-related technologies, the same research group published another article in 1994, in which the concept of ‘eNose’ was proposed and defined in detail^146^. According to these studies, eNose is a biomimetic detection instrument composed of a sensor array that can react with multiple gases, and a specific identification methodology enable the identification and classification of individual or compound gases. The introduction of this concept signaled the transition of eNose technology from a phase of growth period to one of maturity, leading to a stage of steady development. In the same year, the world witnessed the emergence of the first commercial ‘eNose’ instrument.

**Fig. 9:**
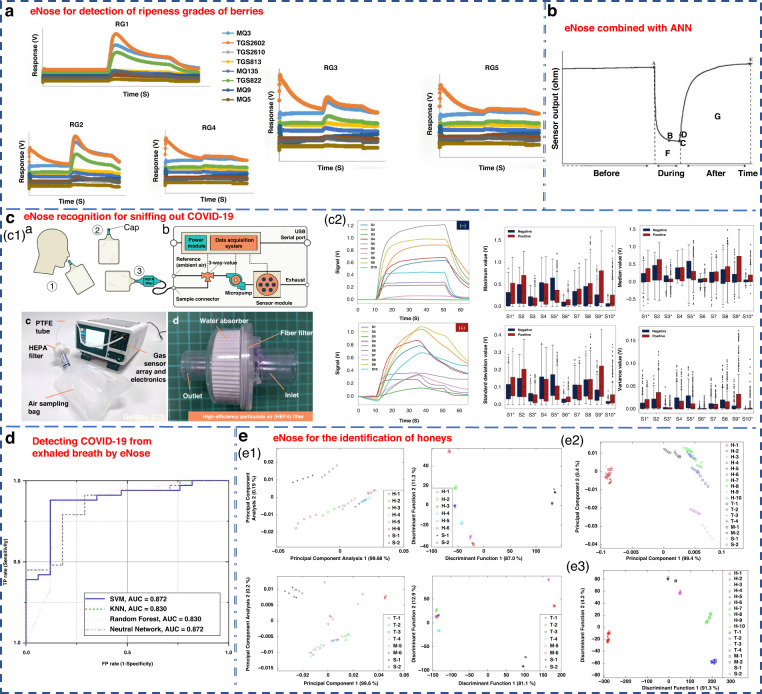
Examples of eNose systems. **a** eNose for the detection of ripeness grades of berries^147^. Copyright 2022 John Wiley & Sons. **b** eNose combined with ANN for the classification of pecorino cheese^148^. Copyright 2011 Elsevier. **c** A rapid noninvasive eNose based on breath-fingerprint recognition for sniffing out COVID-19^152^. Copyright 2022 Springer Nature. **d** Clinical studies of detecting COVID-19 from exhaled breath by eNose^153^. Copyright 2022 Springer Nature. **e** eNose sensor for the identification of different honeys^155^. Separate plot of 32 eNose sensor results (**e1**) for honey assessment by using LDA (**e2**) and PCA (**e3**). Copyright 2011 MDPI

In recent years, due to the continuous development of eNose technology, remarkable progress has been achieved in the food, medicine, agriculture and other light industries. The Nahid group used an eNose system to classify the maturity of berries into five levels in 2020^147^. ANN, PCA and LDA were applied to the recognition mode of the sensor array. Among them, the performance of ANN was the best, achieving a 100% discrimination rate for blackberry and 88.3% for bayberry. PCA achieved discrimination rates of 97% for blackberry and 93% for bayberry, while LDA exhibited the lowest efficacy (Fig. 9a). Cevoli et al. used an eNose equipped with six MOS sensors and ANN methods to successfully classify Italian cheese (Fig. 9b). The final accuracy was 100%^148^.

Machado et al. utilized the Cyranose 320 eNose to analyze the exhaled gas composition of 14 patients with bronchial cancer and 45 H subjects^21^. By combining with SVM, it achieved an accuracy of 72% and specificity of 92% for LC detection. The Cyranose 320 eNose was also used to distinguish NSCLC, COPD, and H control subjects. The results showed that the olfactory characteristics of LC patients could be distinguished from those of COPD patients and H subjects^75^. Horvath et al. utilized an eNose system to distinguish different VOCs produced by ovarian cancer and normal tissues. It obtained a remarkable recognition accuracy of 100% when using 15 samples for each tissue type^149^.

Recently, Wang’s group from Zhejiang University applied an eNose to detect pests during crop storage and early bollworm infestation in cotton^150^. It could effectively distinguish healthy crops from pest-infested crops^151^. Dian et al. developed a rapid noninvasive eNose based on expiratory breath fingerprinting recognition for sniffing out COVID-19^152^. Notably, the eNose system exhibited high levels of systematic detection accuracy (88–95%), sensitivity (86–94%), and specificity (88–95%), as shown in Fig. 9c. These findings indicated the potential of the use of GeNose C19 as a highly effective breath testing device for rapid COVID-19 screening. In a related study, the outcomes of COVID-19 detection within a local hospital were detailed utilizing a developed electronic setup incorporating commercial VOC gas sensors^153^. ROC curves were generated for a cohort of 50 samples, consisting of 33 COVID-19-infected patients and 17 H. Four detection algorithms of SVM, KNN, RF, and neural network, were examined, as illustrated in Fig. 9d.

Chen et al. proposed a novel eNose model based on a virtual array SAW sensor^154^. The image recognition method and improved neural network were utilized to analyze the output response of the sensor. This eNose system successfully detected 11 LC-related marker VOCs and achieved promising diagnostic results in hospitalized patients. Zakaria et al. utilized an eNose system comprising 32 sensors combined with probabilistic neural networks (PNNs) to differentiate honey from various floral sources, pseudo-honey and syrup (Fig. 9e). It was able to compositionally classify different samples with an accuracy of 92.59%^155^.

Through the above research, the emergence of various commercial eNoses and self-developed eNoses have been widely used in various fields. According to the analysis of the literature in recent years, the application of the eNose system in the field of clinical medicine is increasing. In addition to the early cancer screening, bacterial pathogen identification and analysis of superficial wound microorganisms mentioned in the manuscript, several research teams have also developed respiratory tests for COVID-19 in the last three years^156,157^. The Helicobacter pylori breath test is also widely used in clinical practice^158^. The sensors and algorithms complement each other. Based on these test results, the high integration of gas sensor arrays and intelligent algorithms in the future will provide great prospects for the application of eNose systems in the field of respiratory diagnosis.

## Conclusion and perspective

In the pursuit of early diagnosis and timely treatment of diseases, breath testing has gained considerable attention due to its inherent safety, noninvasiveness, and convenience. eNose is capable of providing rapid qualitative or semiquantitative results and considered an ideal device for swift breath screening in disease detection. In this review, a comprehensive examination of gas sensor arrays and pattern recognition algorithms employed in eNose systems that have been widely utilized for expiratory diagnosis in recent years is presented.

The widespread clinical application of eNose systems requires the synchronized advancement of physiological mechanisms and sensing technologies. The primary challenge is achieving selective detection within the complex human exhaled environment while avoiding the impact of other VOCs and humidity. Therefore, it is essential to further improve the selectivity of the eNose system. Furthermore, to ensure their suitability for the human expiratory environment in clinical applications, the influence of high humidity needs to be addressed. This can be accomplished by further exploring potential biochemical and metabolic mechanisms underlying expiratory markers while considering the pathological conditions of patients.

Additionally, the selection of appropriate sensing materials and processing techniques for gas sensors within eNose systems should be guided by the device’s intended purpose and operational requirements. The implementation of targeted pattern recognition algorithms will enable the identification of correlations between the sensor response signals and physiological indicators and can improve the robustness of the exhaled biomarkers for clinical diagnosis. Moving forward, the high integration of gas sensor arrays and intelligent algorithms holds great promise for enhancing the applications of eNose systems in the field of breath diagnosis.
